# MiR-15b-3p weakens bicalutamide sensitivity in prostate cancer via targeting KLF2 to suppress ferroptosis

**DOI:** 10.7150/jca.92379

**Published:** 2024-03-02

**Authors:** Chunlin Zhang, Haitao Yu, Xuesong Bai, Xiang Zhou, Zhenwei Feng, Yang Li, Xiang Peng, Yuhua Mei, Li Li, Xin Gou, Yuanzhong Deng, Guo Chen

**Affiliations:** 1 Department of Urology, The First Affiliated Hospital of Chongqing Medical University, Chongqing, China.; 2 Chongqing Key Laboratory of Molecular Oncology and Epigenetics, Chongqing, China.

**Keywords:** miRNA, Prostate cancer, Ferroptosis, Prostate-specific antigen, Bicalutamide resistance

## Abstract

Bicalutamide (BIC) resistance impedes the treatment of prostate cancer (PCa) and seems to involve ferroptosis; however, the underlying mechanism remains unclear. Our study aimed to explore how miR-15b-3p modulates ferroptosis in response to BIC resistance and determine whether the miRNA is suitable for early screening of PCa. Here, we found that PCa tissues had significantly higher miR-15b-3p expression than adjacent normal tissues. Analysis of blood samples in patients who underwent prostate-specific antigen (PSA) screening revealed that miR-15b-3p was a more accurate diagnostic than PSA (miR-15b-3p area under the curve [AUC] = 0.941, PSA AUC = 0.815). In vitro experiments then demonstrated that miR-15b-3p expression was markedly higher in LNCaP, PC-3, and DU145 cells than in RWPE-1 cells. Treatment with BIC decreased miR-15b-3p expression and progressive ferroptosis. Mechanistically, we identified KLF2 as the downstream target of miR-15b-3p. Overexpressing KLF2 facilitated ferroptosis via augmenting MDA and iron concentrations, in turn inhibiting the SLC7A11/GPX4 axis and decreasing GSH concentration. Through modulating ferroptosis, miR-15b-3p mimic and inhibitor weakened and enhanced BIC sensitivity, respectively. Furthermore, BIC treatment limited xenograft tumor volume in vivo, whereas agomir-15b-3p promoted tumor growth, indicating that miR-15b-3p attenuated the tumor-suppressive effects of BIC. Taken together, our results suggested that miR-15b-3p is crucial to BIC resistance, specifically via targeting KLF2 and thereby suppressing ferroptosis. High miR-15b-3p expression in early PCa screening should reflect a higher probability of cancer. In conclusion, miR-15b-3p has strong potential as a screening and diagnostic biomarker with reliable prospects for clinical application. Furthermore, because patients with high miR-15b-3p and low KLF2 expression have a greater risk of BIC resistance and malignant progression, targeting the miRNA and its downstream protein may be a new treatment strategy.

## Introduction

Prostate cancer (PCa) is the second most common cancer affecting the male population^1^. Approximately 10 million men worldwide are diagnosed with PCa, resulting in over 400,000 deaths annually. By 2040, the mortality rate of PCa is expected to surpass 800,000 deaths^2^, ^3^. The primary risk factors for PCa include smoking, obesity, age, race, and family history^4^. Inadequate screening among older men has led to the cancer being far advanced by the time of diagnosis, severely lowering quality of life^5^. However, early diagnosis of PCa is associated with a high cure rate, emphasizing the need for novel early detection and diagnostic methods.

Prostate-specific antigen (PSA) is the most commonly used prostate-specific biomarker for early PCa screening. However, PSA levels can be elevated in other non-cancerous diseases, such as prostatitis, urinary tract infections, and benign prostatic hyperplasia^6^-^8^, leading to numerous false positives. Furthermore, despite widespread use, the sensitivity of PSA for diagnosing PCa is also insufficient^9^. Currently, PSA is coupled with prostate biopsy^10^. the gold standard of PCa diagnosis that can confirm cancer presence and identify its stage. To undergo prostate biopsy, a criterion of total PSA (tPSA) > 10 ng/mL must be reached, but of these patients, only 25%-30% are diagnosed with PCa^11^, ^12^. For patients in the PSA gray zone (4 ng/mL < tPSA < 10 ng/mL), no biomarkers are available to verify whether a prostate biopsy is required^13^. Therefore, new biomarkers are urgently needed to better guide biopsy decisions.

MicroRNAs (miRNAs) are highly conserved small non-coding RNA, typically 19-24 nucleotides in length; they are indispensable in post-transcriptional mRNA regulation^14^. Through binding to a broad spectrum of target genes, miRNAs inhibit their translation or promote their degradation^15^, ^16^. Furthermore, miRNAs have been implicated in numerous diseases, particularly cancers^17^ including PCa. For example, miRNA-671-5p facilitates proliferation, migration, and invasion by targeting the NFIA/CRYAB axis^18^. Additionally, miR-423-5p can directly interact with MALAT1 to prevent MALAT1-mediated PCa proliferation and metastasis 19. However, although miR-15b-3p has been linked to other cancers, its role in PCa remains unclear. Previous studies on gastric carcinoma found that miR-15b-3p binds to DYNLT1 and activates the Caspase3/9 pathway, thus accelerating tumorigenesis^20^. Additionally, in the serum extracellular vesicles of patients with glioblastoma, miR-15b-3p is upregulated and has potential as a biomarker of poor prognosis^21^. These findings support miR-15b-3p as a carcinogenic molecule, demonstrating the need for further investigation regarding its mechanistic involvement in PCa development, progression, diagnosis, and drug resistance.

A member of the Kruppel-like factor family member, KLF2 (molecular mass = 37 kDa) controls transcription via binding to DNA with its conserved zinc-finger domains^22^. KLF2 exhibits tumor-suppressing effects in various malignancies, including breast cancer^23^, non-small cell lung cancer^24^ and pancreatic cancer^25^. In breast cancer, KLF2 is a diagnostic/prognostic biomarker because it is downregulated in tumor tissues compared with para-carcinoma controls. Mechanistically, KLF2 activates dendritic cells to modulate the immune microenvironment of breast cancer and restrains angiogenesis via downregulating VEGFA and HIF1α expression^23^. In lung carcinoma, KLF2 inhibits metastasis by regulating occludin expression and the VEGF/MMP pathway, limiting vascular permeability and angiogenesis^24^. Furthermore, KLF2 cooperates with FOXO4 to promote p21 expression and inhibit the migration and proliferation of pancreatic carcinoma cells^25^.

In contrast, detailed research on KLF2 action in PCa has not been conducted. Studies have implicated the protein in ferroptosis, a newly identified type of cell death that is directly linked to lipid peroxidation^26^ and cancer cell biology. In gastric cancer, CST1 acts through OTUB1 to regulate GPX4 stability, in turn, inhibiting ferroptosis and promoting metastasis^27^. Tumors with high TYRO3 expression have inhibited ferroptosis, which can lead to anti-PD-1/PD-L1 resistance^28^. Finally, in clear cell renal cell carcinoma, KLF2 deficiency inhibits ferroptosis by impairing GPX4 transcriptional repression, promoting tumor migration and invasion^29^. However, KLF2 modulation of ferroptosis has not been demonstrated in PCa.

Bicalutamide (BIC) is a nonsteroidal anti-androgen widely used as a first-line clinical treatment for advanced PCa 30. However, while BIC is effective in the initial stages of treatment, resistance often develops and eventually leads to castration-resistant PCa (CRPC)^30^, ^31^. Therefore, the exploration of BIC resistance mechanisms is urgent and clinically important. MiR-15b-3p may play a role in BIC resistance, but the exact involvement remains unclear.

In this study, we investigated whether miR-15b-3p can be a new diagnostic biomarker for PCa. Our findings revealed that the positive diagnostic rate is far higher with miR-15b-3p than with tPSA. We also identified a novel mechanism that inhibits ferroptosis in PCa, specifically the direct interaction between miR-15b-3p and KLF2 3ʹUTR. Moreover, we confirmed that miR-15b-3p participates in BIC resistance. Our study provides novel insight into the diagnosis and treatment of PCa.

## Materials and Methods

### Clinical blood and tissue sample

Between December 2020 and December 2022, 570 blood samples were collected for PSA screening during the implementation of a project on PCa screening and intervention in Chengdu and Chongqing. Between February 2023 and January 2024, 34 PCa tumors and adjacent normal tissues were collected from patients who underwent tumor excision surgery at our hospital. These tissues were immediately immersed in liquid nitrogen until protein or RNA was extracted.

### Participants in PCa screening

Male patients involved in PCa screening were selected from 47 hospitals in Chongqing and Sichuan, China. Inclusion criteria were as follows: (1) good physical condition with a life expectancy of >10 years; (2) over age 50, over age 45 with a family history of PCa, or over age 40 with a baseline PSA > 1 µg/L (high-risk individuals).

### Cell culture and treatment

The RWPE-1 and PCa cell lines (LNCaP, PC-3, and DU145) were purchased from Shanghai Zhong Qiao Xin Zhou Biotechnology (Shanghai, China). LNCaP and PC-3 cells were cultured in RPMI-1640 medium (Gibco, USA) supplemented with 10% fetal bovine serum (FBS; Procell, Wuhan, China). RWPE-1 and DU145 cells were cultured in a special medium purchased from Shanghai Zhong Qiao Xin Zhou Biotechnology. All cell lines were incubated at 37℃ in a humidified cell incubator set to 5% CO2. Bicalutamide concentrations ranged from 2.5 to 12.5µM.

### Oligonucleotides, plasmids and cell transfection

Full-length KLF2 cDNA was subcloned into pcDNA3.1. Mimi NC, miR-15b-3p mimic, and miR-15b-3p inhibitor were synthesized by Tsingke Biotechnology. The miR-15b-3p mimic sequences were sense-CGAAUCAUUAUUUGCUGCUCUA and antisense-GAGCAGCAAAUAAUGAUUCGUU. The miR-15b-3p inhibitor sequence was UAGAGCAGCAAAUAAUGAUUCG. Plasmids were validated with DNA sequencing, and oligonucleotides were transfected using Lipofectamine 3000 (Invitrogen).

### Quantitative real-time PCR (qRT-PCR)

Total RNA was extracted from preprocessed cells and clinical tissues using TRIzol reagent (Abclonal, China), and from whole blood using a whole-blood RNA isolation kit (Simgen, China). Purified RNA was reverse-transcribed using a PrimeScript qRT-PCR kit (Abclonal, China). Next, qRT-PCR was performed using a SYBR(R) Prime-Script qRT-PCR kit (Abclonal, China) on an ABI 7500 Real-Time PCR System (Applied Biosystems, USA). Relative expression was calculated with the 2^-ΔΔCt^ method.

### Colony formation assay

Processed LNCaP cells were seeded at 1500 cells/well in a six-well plate and incubated until colonies were visible. Cultured cells were then fixed with 4% paraformaldehyde and stained with 0.1% crystal violet. Colonies were counted in ImageJ.

### Lipid peroxidation, iron and glutathione assay

The assay was performed following published methods^32^. Malondialdehyde (MDA) was quantified using a lipid peroxidation assay kit (Abcam, UK). After adding TBA to standards and samples, the mixtures were incubated at 95°C for 1 h, followed by a 10 min ice bath. Absorbance was measured at 532 nm using a microplate reader.

Iron was quantified with an Iron Assay kit (ElabScience). Homogenized samples in iron assay buffer were centrifugated at 16,000 ×g and 4°C for 10 min. The supernatant (10 µL) was mixed with 90 µL iron assay buffer and then incubated with 5 µL iron reducer at 25°C for 30 min. Finally, each mixture was incubated with an iron probe in the dark, and absorbance was measured at 532 nm.

Glutathione (GSH) was measured using a commercial glutathione assay kit (Elabscience, CHN). A 5% 5-sulfosalicylic acid solution was used to prevent GSH autoxidation and degradation. After lysing, 10 µL of the supernatant was incubated with the reaction mix. Finally, GSH content was determined by measuring absorbance at 405 nm.

### Cell counting kit-8

Transfected LNCaP cells (3 × 103 per well) were inoculated into a 96-well plate with 200 µL RPMI-1640 medium containing 10% FBS. Next, 10 µL of CCK-8 reagent was added to each well and incubated at 37°C for 2 h. Absorbance was measured at 450 nm using a microplate reader.

### Luciferase reporter assay

The 3ʹ-UTR KLF2 sequence containing the miR-15b-3p binding site and the mutant 3ʹ-UTR KLF2 sequence were subcloned into the pmirGLO vector. Next, 293T cells were co-transfected with luciferase reporters plus the miR-15b-3p mimic (agomir) or negative control. Luciferase activity was assessed with the Dual-Luciferase® Reporter Assay System (Promega, USA).

### Western blotting (Wb)

Total protein was extracted from cells and tissues using phenylmethanesulfonyl fluoride (PMSF) and RIPA lysis buffer (Beyotime) at 1:100. Proteins were separated using SDS-PAGE, and then transferred to PVDF membranes (EMD Millipore). After blocking in Tris-buffered saline (TBS) containing 5% skim milk, membranes were incubated overnight with primary antibodies for SLC7A11 (Zenbio, R26116), GPX4 (Zenbio, 381958), KLF2 (Abcam, ab194486), and β-actin (Proteintech, 66009-1-Ig) at 4°C. On the next day, membranes were incubated with a secondary antibody for 1 h at room temperature. Protein blots were visualized using enhanced chemiluminescence (Cell Signaling Technology, USA).

### Cell line-derived xenografts

Seven-week-old male nude mice were randomly divided into four groups (n = 3/group) and subcutaneously injected with LNCaP cells (1 × 106/100 μL) at the root of the right thigh. After tumor formation, miRNA-NC and miRNA-agomir were injected intratumoral every 3 days, while BIC was intraperitoneally injected every 4 days. Tumor volume (1/2 × length × width2) and mouse weight were monitored for 3 weeks.

### Immunofluorescence

Xenograft sections derived from LNCaP cell lines were stained with anti-Ki67 antibody overnight at 4°C. They were then incubated with a secondary antibody (Alexa Fluor 488 Conjugate) for signal visualization. Nuclei were stained with DAPI (Beyotime, China). A laser confocal microscope (Leica Microsystems AG) was used to acquire images.

### Statistical analysis

Receiver operating characteristic (ROC) curves were generated to evaluate the accuracy of PSA and miR-15b-3p in PCa diagnosis. The area under the curve (AUC) was calculated using a ROC package.

Data were presented as mean ± SD of observations made in triplicate. Statistical tests were run in GraphPad Prism 9.5.1 and SPSS version 21.0. Normality and homogeneity of variance were tested before conducting one-way ANOVA, t-tests, and Pearson's correlations. Significance was set at P < 0.05.

## Results

### MiR-15b-3p is more accurate for early PCa screening than PSA

**Figure 1:**
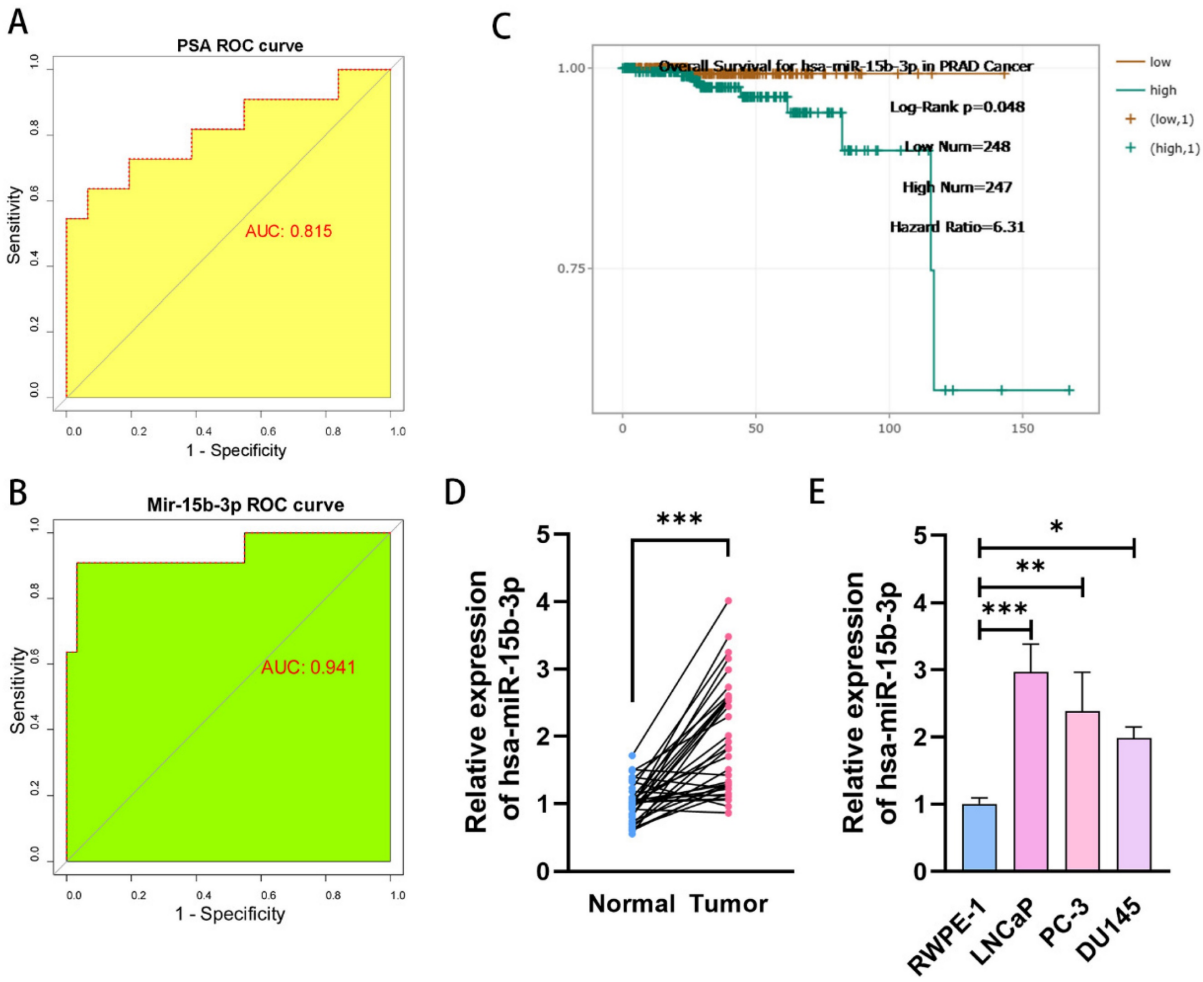
** MiR-15b-3p as a prostate-specific biomarker for PCa early screening is more accurate than PSA.** (A, B) The different AUC values between PSA and miR-15b-3p of ROC curves for PCa early screening. (C) The overall Survival curves for has-miR-15b-3p in PCa. (D) MiR-15b-3p expression levels in 34-surgically resected human prostate tumor samples and their adjacent normal tissues. (E) MiR-15b-3p expression levels in LNCaP, PC-3 and DU145 were higher than RWPE-1. (All results are three distinct repetitions. ***p<0.001, **p < 0.01 and *p < 0.05 represent significant differences between two groups).

We conducted a comprehensive PSA screening program for 3 years to identify potential patients with PCa in Chongqing and Sichuan. In total, 16,746 men underwent PSA screening. Among them, 1,005 cases (6%) were screened out for abnormal PSA levels (median PSA = 6.10 ng/mL, range: 4.0-102 ng/mL). Our center tested 570 patients and identified 52 (9.12%) with abnormal PSA levels. From these 52, 42 (80.77%) consented to prostate biopsy, and 11 were eventually diagnosed with PCa (Supplementary file). Therefore, we can conclude that PSA-based PCa screening has a positivity rate of only 26.19% (11/42). We then drew a ROC curve analyzing PCa screening results and calculated an AUC of 0.815 (Figure 1A). Next, we quantified serum miR-15b-3p expression for all 42 cases using qRT-PCR (Table S1). The AUC of miR-15b-3p-based screening was 0.941 (Figure 1B). In support of this strong effectiveness, estimations using StarBase, a miRNA survival analysis website, revealed that high miR-15b-3p expression was associated with worse overall survival (Figure 1C).

Next, we compared miR-15b-3p expression between 34 surgically resected prostate tumors and adjacent normal tissues. Tumor miR-15b-3p expression was higher than expression in normal tissues (Figure 1D). Similar results were obtained in vitro (Figure 1E).

### BIC inhibits PCa cell viability by inducing ferroptosis

**Figure 2:**
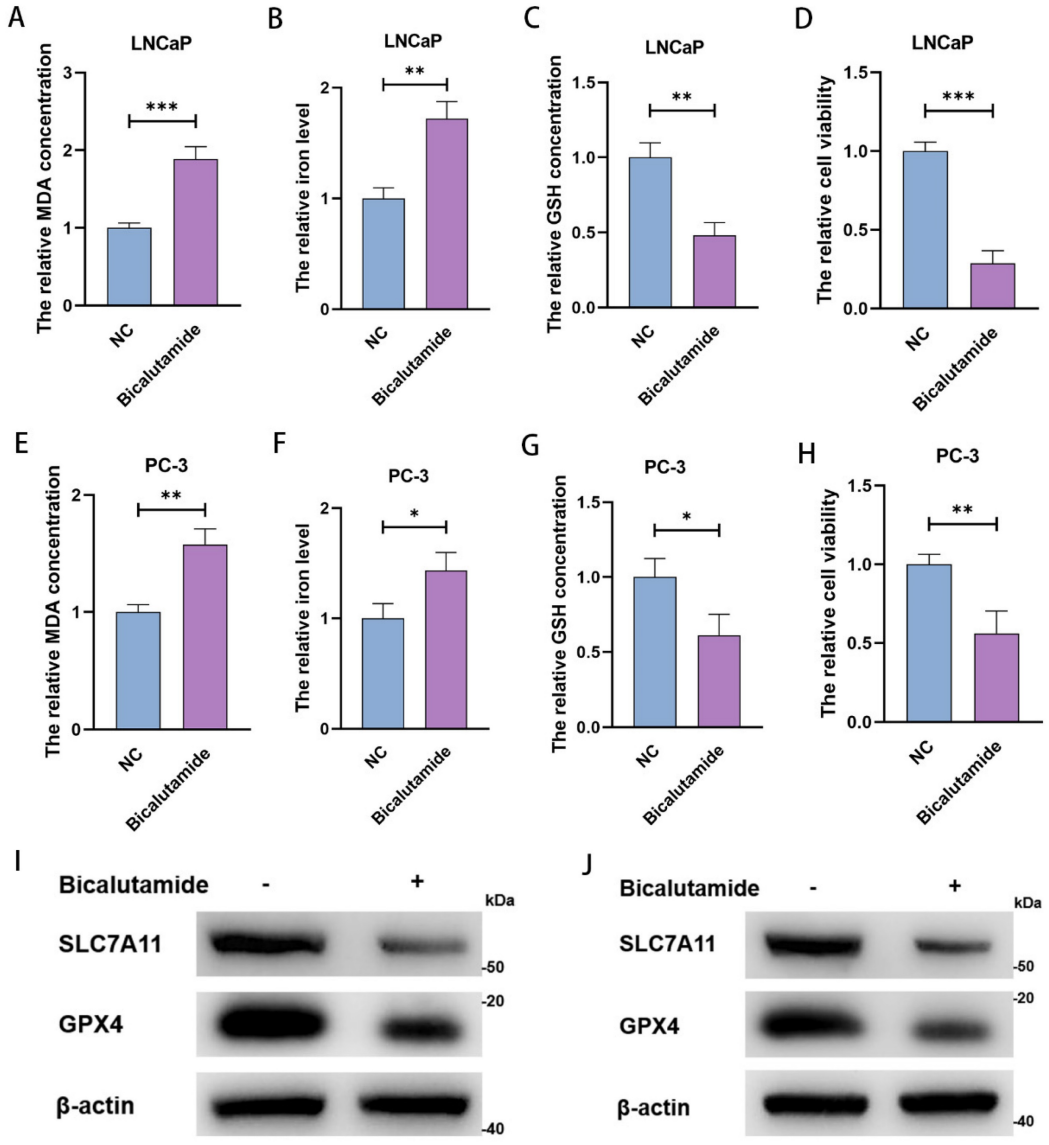
** BIC inhibits the PCa cells viability in the form of induction of ferroptosis.** (A-D) MDA concentration, iron level, GSH concentration and cell viability were measured in LNCaP cells after 10µM BIC treatment for 48 hours. (E-H) MDA concentration, iron level, GSH concentration and cell viability were measured in PC-3 after 15µM BIC treatment. (I, J) SLC7A11 and GPX4 were tested after BIC treatment in LNCaP and PC-3. (All results are three distinct repetitions. ***p<0.001, **p < 0.01 and *p<0.05 represent significant differences between two groups).

After treating LNCaP and PC-3 with 10 µM and 15 µM BIC for 48 h, we measured MDA concentration, iron level, GSH concentration, and cell viability. As expected, we observed significant increases in MDA concentration and clustering of iron levels (Figure 2A, 2B, 2E, and 2F), as well as notably lower GSH concentration (Figure 2C and 2G). These changes are prerequisites for ferroptosis. Moreover, BIC treatment significantly decreased cell viability (Figure 2D and 2H). As further confirmation, we observed decreased SLC7A11 and GPX4 levels (Figure 2I and 2J). These results strongly suggest that the mechanism underlying BIC action is inducing ferroptosis in PCa cells.

### MiR-15b-3p suppresses ferroptosis to weaken BIC sensitivity in PCa

**Figure 3:**
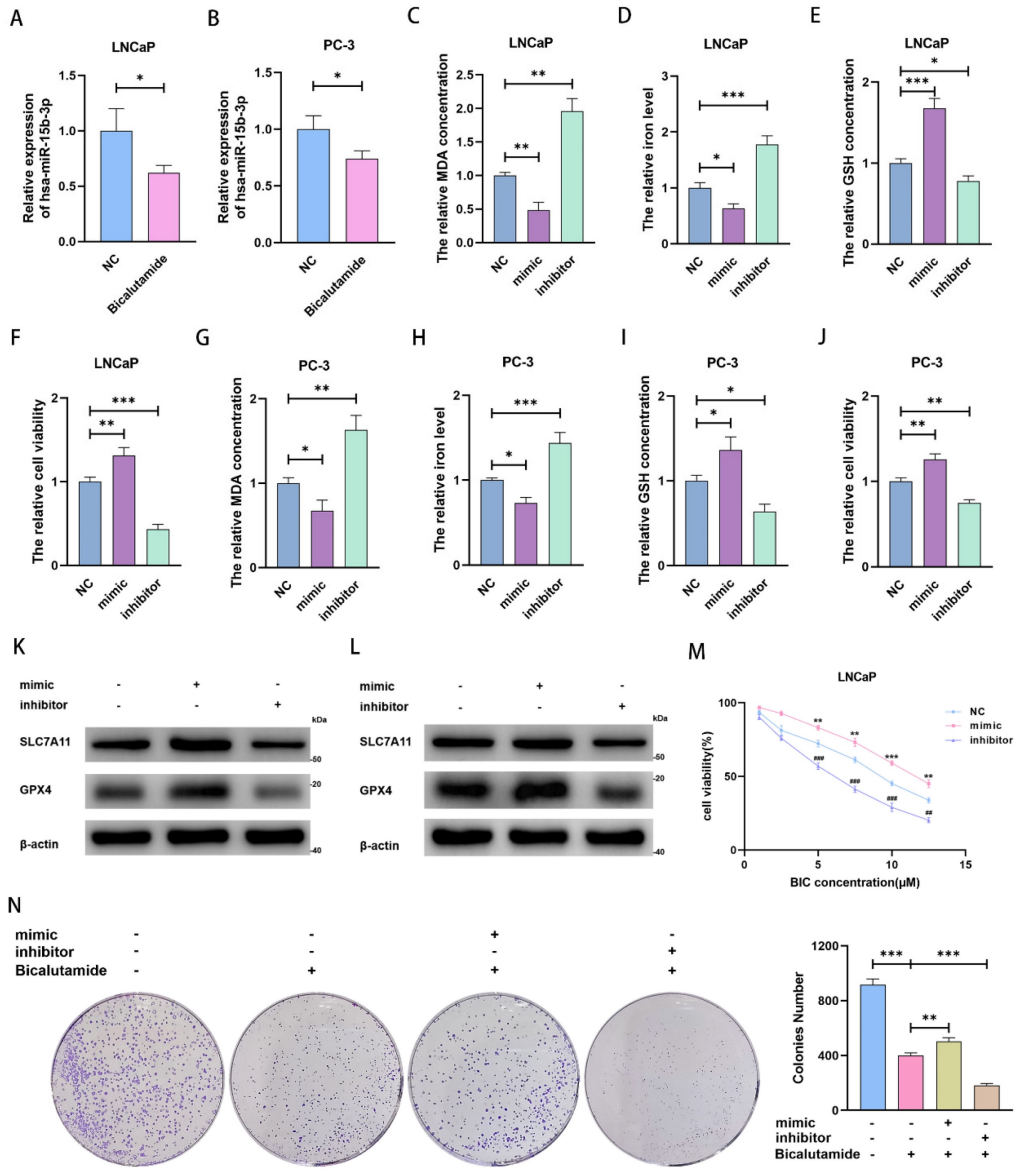
** MiR-15b-3p suppresses ferroptosis for weakening BIC sensitivity in PCa.** (A, B) miR-15b-3p was decreased by BIC treatment. (C-J) MDA concentration, iron level, GSH concentration and cell viability were measured after transfection of has-miR-15b-3p mimic and inhibitor for 24 hours in LNCaP and PC-3. (K, L) SLC7A11 and GPX4 were regulated after transfection of has-miR-15b-3p mimic and inhibitor for 48 hours in LNCaP and PC-3. (M) mimic and inhibitor regulated the BIC sensitivity in LNCaP by CCK8. (N) LNCaP clone formation for transfection of has-miR-15b-3p mimic and inhibitor under BIC treatment. (All results are three distinct repetitions. ***p<0.001, **p < 0.01, *p < 0.05, ###p<0.001 and ##p < 0.01 represent significant differences between two groups).

We noticed that miR-15b-3p expression was downregulated after BIC treatment (Figure 3A and 3B). Considering that miR-15b-3p is highly expressed in PCa and that ferroptosis is repressed in most cancer cells, we speculated that miR-15b-3p may inhibit ferroptosis in PCa. To test this hypothesis, we transfected the hsa-miR-15b-3p mimic and inhibitor into LNCaP and PC-3 cells (Figure S1A and S1B). Subsequently, we demonstrated that miR-15b-3p overexpression hampered MDA accumulation and downregulated intracellular iron levels. Downregulating miR-15b-3p incurred the opposite results (Figure 3C, 3D, 3G, and 3H). In addition, mimic treatment increased GSH concentration and enhanced cell viability, while inhibitor treatment reversed these effects (Figure 3E, 3F, 3I, and 3 J). Furthermore, the miR-15b-3p inhibitor facilitated ferroptosis via inhibiting SLC7A11 and GPX4 in LNCaP and PC-3 cells, whereas the mimic upregulated both proteins (Figure 3K and 3L). These results indicated that miR-15b-3p blocks ferroptosis in PCa cells and promotes cell viability.

To verify the importance of miR-15b-3p in BIC treatment, we tested cells with a BIC concentration gradient. We observed that the miR-15b-3p mimic and inhibitor weakened and enhanced the effects of BIC, respectively (Figure 3M and Figure S1C). Moreover, the mimic partially rescued BIC-induced inhibition of clone formation, whereas the inhibitor accelerated it (Figure 3N).

### KLF2 is the downstream target of miR-15b-3p in PCa

**Figure 4:**
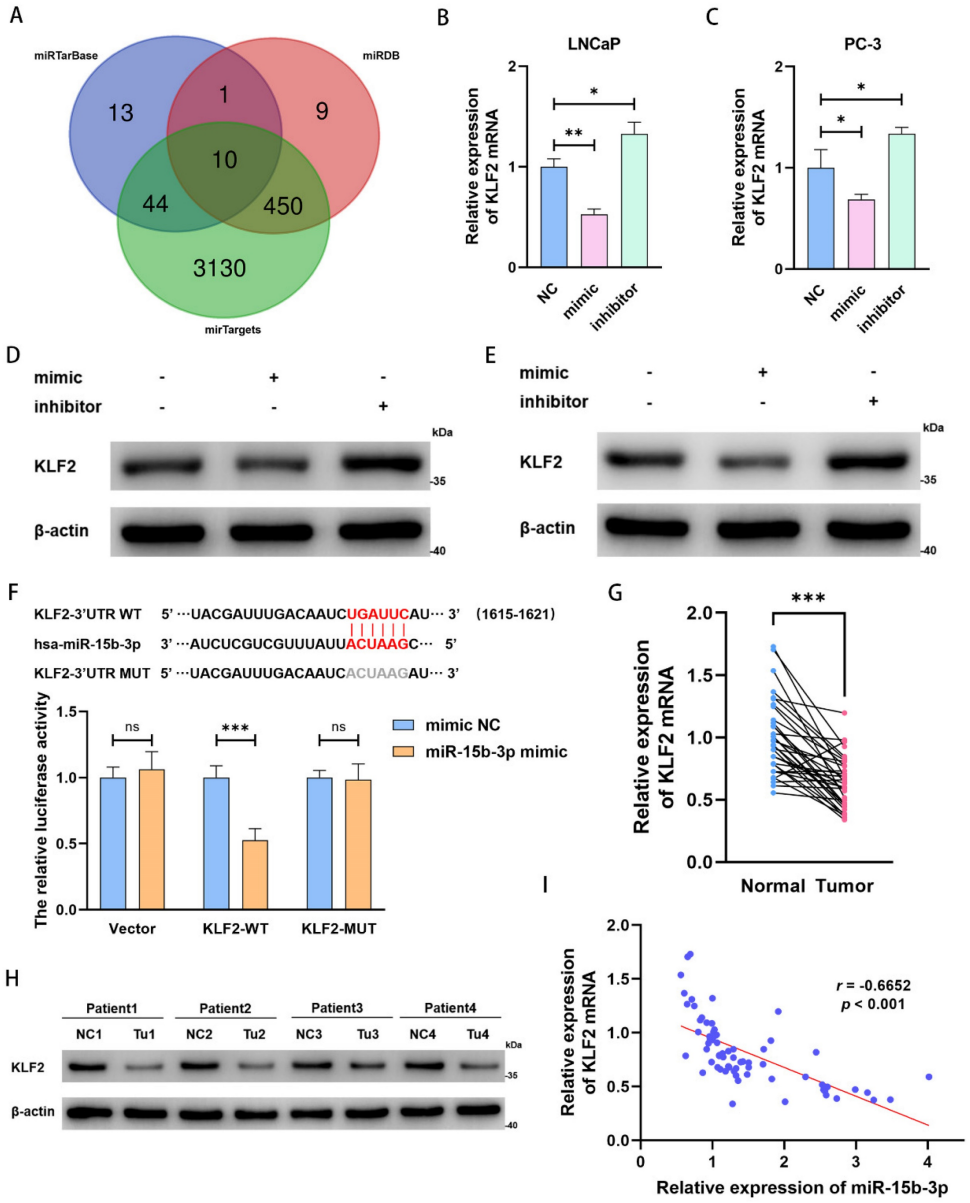
** KLF2 is the downstream target of miR-15b-3p in PCa.** (A) Online predicting the miR-15b-3p target genes. Venn gram showed the intersection genes. (B, C) KLF2 mRNA levels after transfection of has-miR-15b-3p mimic and inhibitor in LNCaP and PC-3. (D, E) KLF2 proteins levels after transfection of has-miR-15b-3p mimic and inhibitor in the above two cell lines. (F) Online predicting of the interaction sites between miR-15b-3p and KLF2, and luciferase reporter assays demonstrated KLF2 was a direct target of miR-15b-3p. (G, H) KLF2 mRNA and protein levels of 34-surgically resected human prostate tumor samples and their adjacent normal tissues. (I) Person correlation analysis between miR-15b-3p and KLF2 in the 34-surgically resected cases. (All results are three distinct repetitions. ***p<0.001, **p < 0.01 and *p < 0.05 represent significant differences between two groups).

To clarify the downstream regulatory mechanism of miR-15b-3p, we predicted target genes in miRTarBase, miRDB, and mirTargets. Based on overlap across the three databases, we obtained 10 predicted target genes (Figure 4A). Among these candidate genes, KLF2 expression significantly decreased after mimic treatment (Figure S1D). Therefore, we concluded that KLF2 is a target of miR-15b-3p.

Next, miR-15b-3p mimic and inhibitor down- and upregulated KLF2 mRNA expression in both LNCaP and PC-3 cells, respectively (Figure 4B and 4C). The same pattern occurred for KLF2 protein levels (Figure 4D and 4E). We then confirmed the direct interaction of miR-15b-3p with KLF2 mRNA 3ʹ-UTR via a luciferase assay. Co-transfection with miR-15b-3p and wild-type KLF2 significantly lowered luciferase activity, whereas co-transfection with miR-15b-3p and mutant KLF2 did not. Therefore, we applied luciferase reporter assays to determine KLF2 binding specificity with miR-15b-3p and demonstrated that the miRNA is a negative regulator of KLF2 expression (Figure 4F).

Because miR-15b-3p expression was higher in PCa tissue than in adjacent normal tissues (Figure 1D), we also checked for similar patterns in KLF2 expression. As expected, KLF2 expression was significantly lower in tumors than in normal tissues (Figure 4G and 4H).

We then analyzed the relationship between miR-15b-3p and KLF2 in 34 surgically resected samples and found a negative correlation (Pearson' r = -0.6652) (Figure 4I). Therefore, KLF2 may be a prostate tumor suppressor gene, and its downregulation is likely due to aberrant miR-15b-3p overexpression. The resultant inhibition of ferroptosis then at least partially promotes PCa cell viability.

### MiR-15b-3p suppresses ferroptosis and spurs BIC resistance in PCa via targeting the KLF2/SLC7A11/GPX4 axis

**Figure 5:**
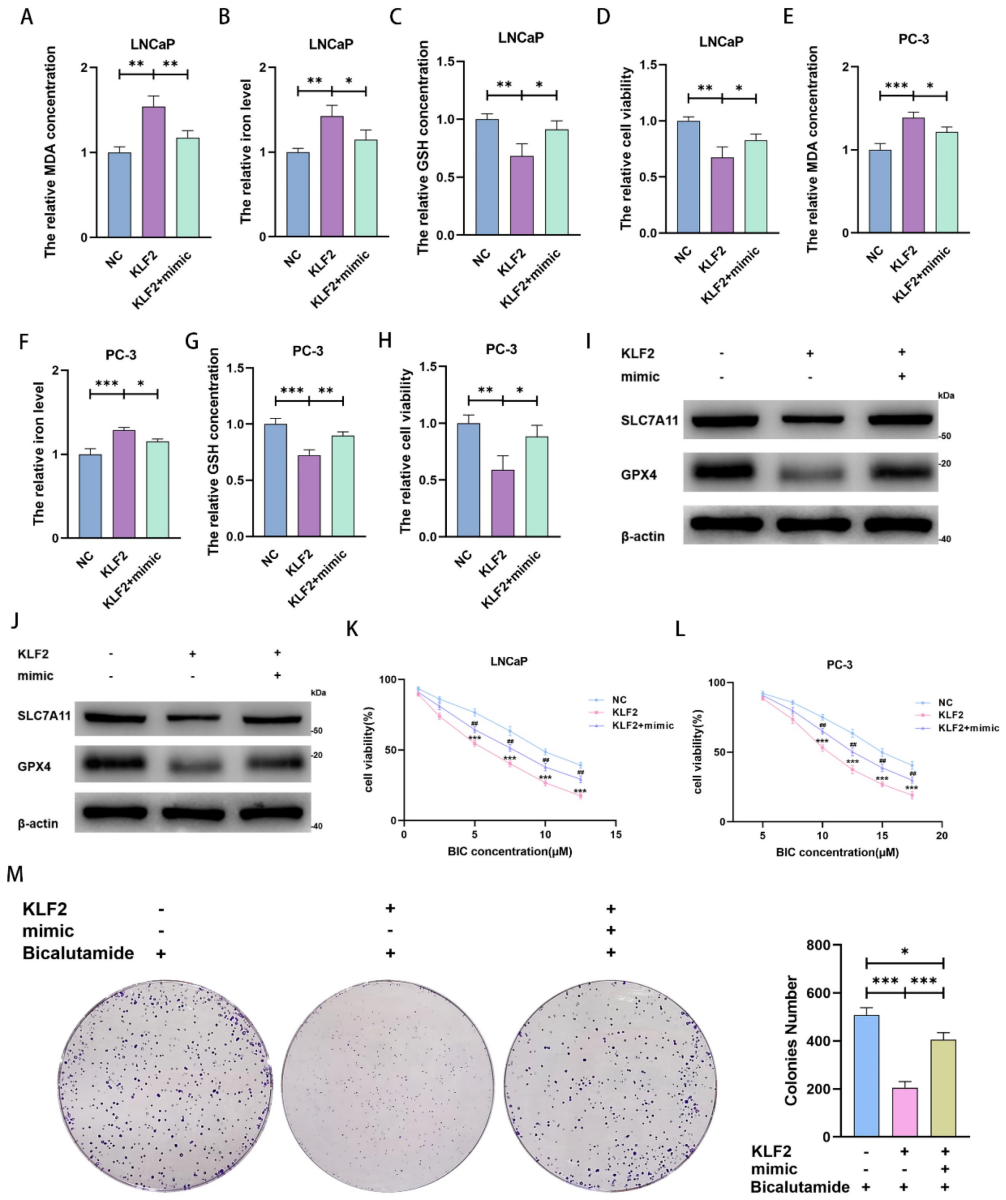
** MiR-15b-3p suppresses ferroptosis and weakens BIC sensitivity by targeting KLF2/SLC7A11/GPX4 axis in PCa.** (A-H) MDA concentration, iron level, GSH concentration and cell viability were measured in KLF2 overexpression LNCaP and PC-3 cells or after transfection of has-miR-15b-3p mimic. (I, J) SLC7A11 and GPX4 levels were detected in KLF2 overexpression LNCaP and PC-3 cells or after transfection of has-miR-15b-3p mimic. (K, L) KLF2 enhanced the BIC sensitivity and mimic rescued it in the above two cell lines. (M) KLF2 inhibited the clone formation and mimic rescued it under BIC treatment. (All results are three distinct repetitions. ***p < 0.001, **p < 0.01, *p < 0.05 and ###p<0.001 represent significant differences between two groups).

We next explored and verified the mechanisms behind miR-15b-3p modulation of ferroptosis. Upon KLF2 overexpression, MDA concentration and iron levels increased, whereas GSH concentration and cell viability decreased. Transfecting the miR-15b-3p mimic after KLF2 overexpression rescued MDA concentration, iron level, GSH concentration, and cell viability in the two cell lines (Figure 5A-5H). Mechanistically, KLF2 overexpression inhibited SLC7A11 and GPX4 expression, accelerating ferroptosis (Figure 5I and 5J). The miR-15b-3p mimic then restored SLC7A11 and GPX4 expression through targeting KLF2 (Figure 5I and 5J). In summary, KLF2 facilitates ferroptosis in PCa, while miR-15b-3p blocks KLF2 activity and thus promotes PCa cell viability.

We also investigated the ability of KLF2 to regulate BIC sensitivity. KLF2 overexpression reinforced the BIC-induced decrease in cell viability, whereas the miR-15b-3p mimic partially erased this decrease (Figure 5K and 5L). In addition, KLF2 overexpression enhanced BIC-induced inhibition of clone formation, while the miR-15b-3p mimic again partially restored clone formation (Figure 5M).

### MiR-15b-3p promotes tumor proliferation and weakens BIC sensitivity in vivo

**Figure 6:**
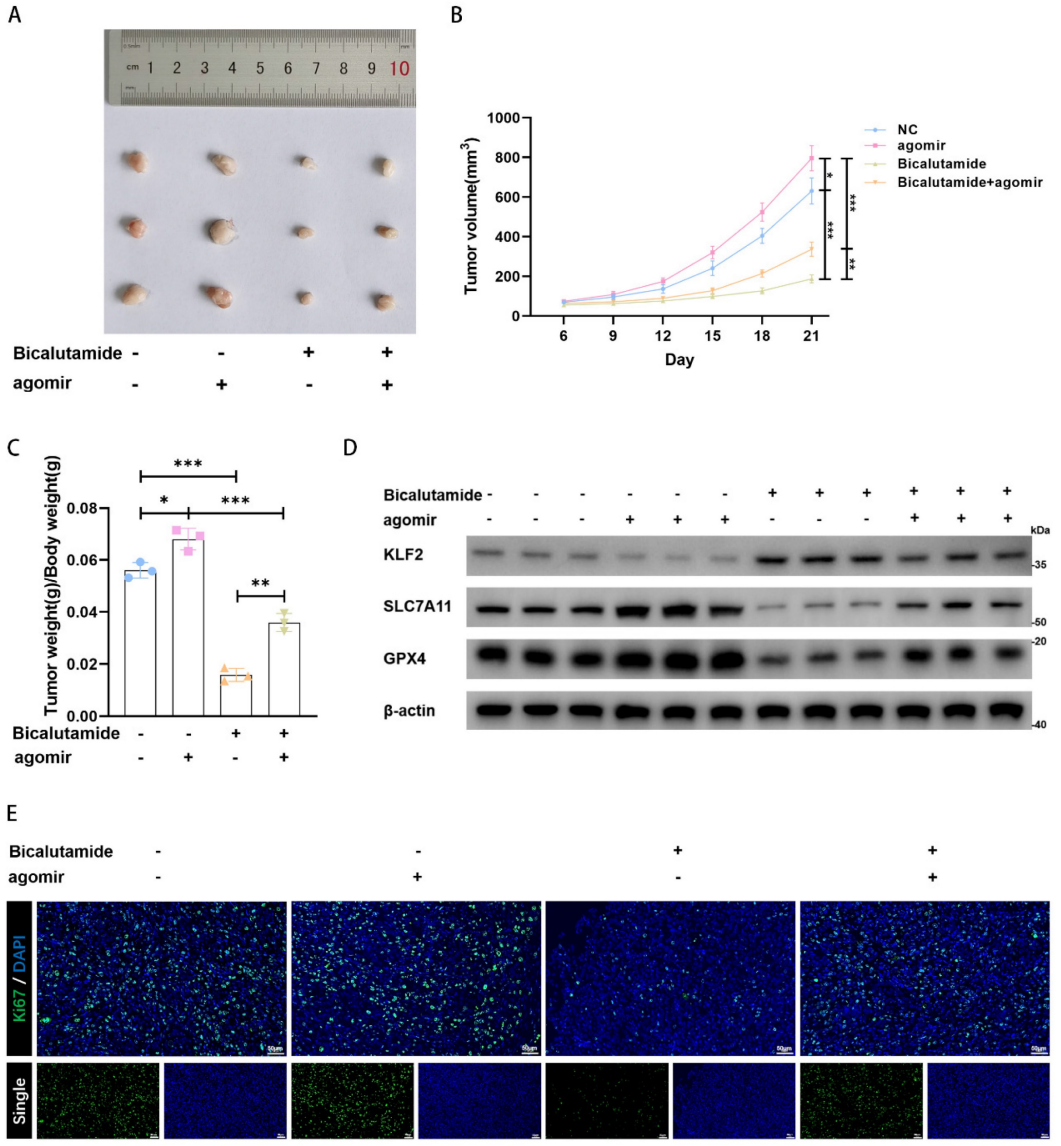
** MiR-15b-3p promotes tumor proliferation and weakens BIC sensitivity in vivo.** (A-C) The tumor volume was recorded for 21 days and then collected for weighing and photoing. (D) KLF2, SLC7A11 and GPX4 were detected in vivo. (E) The ki67 levels in each group, scale bar: 50µm. (All results are three distinct repetitions. ***p<0.001, **p < 0.01 and *p < 0.05 represent significant differences between two groups).

We established LNCaP cell-line-derived xenografts to verify the role of miR-15b-3p in vivo. We discovered that BIC strongly inhibited tumor growth and that the miR-15b-3p agomir promoted tumor growth. Even under BIC treatment, miR-15b-3p rescued tumor growth (Figure 6A, 6B, and 6C). Therefore, miR-15b-3p can attenuate tumor sensitivity to BIC. We also found in vivo evidence that the miR-15b-3p agomir negatively regulated KLF2 expression. While BIC increased KLF2 expression, the miR-15b-3p agomir partially inhibited BIC-induced increase in KLF2 (Figure 6D). Additionally, SLC7A11 and GPX4 levels were negatively correlated with KLF2 (Figure 6D). Lastly, BIC treatment significantly lowered ki67 levels, whereas the miR-15b-3p agomir restored ki67 (Figure 6E). In summary, these results provided in vivo verification that miR-15b-3p attenuates BIC sensitivity by suppressing ferroptosis.

## Discussion

Prostate cancer is the second most common form of cancer affecting men of all ethnicities, ages, and backgrounds^33^. Many patients with PCa are diagnosed using prostate biopsy and pathological analyses^34^. Unfortunately, current screening methods for detecting early disease lack sufficient sensitivity and specificity, leading to unnecessary biopsies and overtreatment^35^. In particular, the use of PSA for population screening has been controversial since the protein was first purified^36^. Similarly, our screening data confirmed the low diagnostic accuracy of PSA. Here, we report for the first time that miR-15b-3p is promising as a novel diagnostic biomarker of PCa. More research across multiple centers is necessary to verify the results observed in our limited number of cases.

The role of miRNAs in PCa has been extensively explored. For instance, miR-205 downregulates cholesterol biosynthesis in aggressive PCa^37^. Likewise, the circSPON2/miR-331-3p axis modulates PRMT5 and epigenetically regulates CAMK2N1 transcription, leading to PCa progression^38^. However, until our study, miR-15b-3p's mechanism of action in PCa had not been investigated. Here, by comparing miR-15b-3p expression across prostate tumors and adjacent normal tissues, we verified that the miRNA is highly expressed in PCa. Survival analysis of miR-15b-3p using starBase also supports our hypothesis that miR-15b-3p promotes PCa. Corroborating our research on the carcinogenic nature of miR-15b-3p, this miRNA is highly expressed in gastric cancer cell lines, tissues, and serum; moreover, exosomal transfer of miR-15b-3p accelerates tumorigenesis and malignant transformation in gastric cancer^20^.

We also successfully predicted the potential target genes of miR-15b-3p, with KLF2 being the most notable candidate in PCa. Interestingly, KLF2 has a suppressive function in many cancers, including PCa, implying that miR-15b-3p promotes PCa progression by inhibiting KLF2. We hypothesized that KLF2 must facilitate ferroptosis because recent studies on clear cell renal cell carcinoma and colorectal cancer have reported that KLF2 induces this form of cell death via the GPX4 and PI3K/AKT pathways^29^, ^39^. Our findings strongly support this hypothesis.

Although BIC — a first-line anti-androgen medication for PCa^40^ — is therapeutically effective, resistance to the drug develops readily. One possible BIC-resistance mechanism is the overamplification of the androgen receptor (AR) gene. The resultant androgen hypersensitivity in PCa cells renders BIC ineffective at blocking the AR signaling pathway^41^. However, until the work we reported here, no clear understanding existed of the role of miRNAs in BIC resistance. Our analysis demonstrated that an miR-15b-3p mimic partially limited BIC sensitivity, whereas the inhibitor increased BIC sensitivity. Based on these findings, we surmised that miR-15b-3p facilitates BIC resistance by suppressing ferroptosis in PCa.

In conclusion, our study revealed that miR-15b-3p negatively regulates its downstream target KLF2 to inhibit ferroptosis in PCa via the SLC7A11/GPX4 axis. The elevated miR-15b-3p expression in PCa cell lines and patient serum provides valuable evidence supporting miR-15b-3p as a promising biomarker for cancer screening and diagnosis. We suggest that miR-15b-3p has considerable clinical significance and is crucial to the development of improved PCa therapies.

## Supplementary Material

Supplementary figure and table.
