# Supplementary material for: MiR-15b-3p weakens bicalutamide sensitivity in prostate cancer via targeting KLF2 to suppress ferroptosis
Source: J Cancer. 2024 Mar 2;15(8):2306–17. doi: 10.7150/jca.92379 (PMC10937263; doi:10.7150/jca.92379)
Supplement: Supplementary file 1 — Supplementary figure and table. [file jcav15p2306s1.pdf]

Figure S1

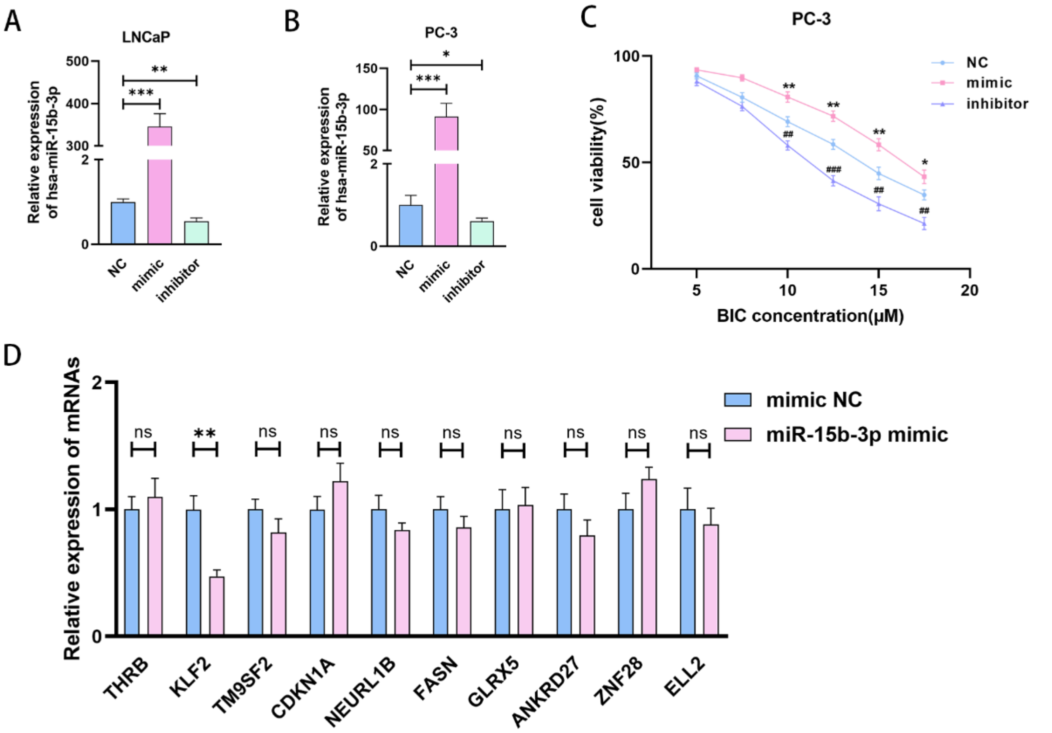

(A, B) Transfection of has-miR-15b-3p mimic and inhibitor were effective in LNCaP and PC-3 cell lines. (C) mimic and inhibitor regulated the BIC sensitivity in PC-3 cells by CCK8. ** and *** represent significant differences between NC and mimic groups; ## and ### represent significant differences between NC and inhibitor groups. (D) mRNAs level of intersection genes after transfection of has-miR-15b-3p mimic.

Table S1. tPSA and miR-15b-3p value of the first affiliated hospital of Chongqing medical university patients

Patients	tPSA(ng/mL)	miR-15b-3p	Diagnosis
**1**	5.41	0.012689	non-PCa
**2**	5.74	0.003965	non-PCa
**3**	8.41	0.006653	non-PCa
**4**	8.23	0.008887	non-PCa
**5**	4.86	0.003364	non-PCa
**6**	8.03	0.006268	non-PCa

**7**	7.48	0.012678	PCa
**8**	6.93	0.000247	non-PCa
**9**	8.63	0.019027	PCa
**10**	6.12	0.001663	non-PCa
**11**	5.88	0.004746	non-PCa
**12**	4.99	0.004008	non-PCa
**13**	6.16	0.003204	non-PCa
**14**	4.59	0.005120	non-PCa
**15**	4.85	0.002187	non-PCa
**16**	8.77	0.009248	non-PCa
**17**	7.43	0.002679	non-PCa
**18**	6.26	0.004903	non-PCa
**19**	5.95	0.000986	non-PCa
**20**	8.02	0.005818	non-PCa
**21**	9.21	0.000776	non-PCa
**22**	6.88	0.005421	non-PCa
**23**	5.5	0.018933	PCa
**24**	7.25	0.008411	non-PCa
**25**	8.19	0.005594	non-PCa
**26**	5.77	0.008657	non-PCa
**27**	9.05	0.000436	non-PCa
**28**	8.08	0.005055	non-PCa
**29**	6.7	0.014621	PCa
**30**	7.37	0.002807	non-PCa
**31**	6.04	0.004306	non-PCa
**32**	5.59	0.006922	non-PCa
**33**	15.9	0.005945	non-PCa
**34**	38.95	0.003232	non-PCa
**35**	37.99	0.000392	non-PCa
**36**	39.44	0.012811	PCa
**37**	39.58	0.015106	PCa
**38**	19.29	0.012691	PCa
**39**	45.88	0.010297	PCa
**40**	49.44	0.017719	PCa
**41**	56.8	0.004034	PCa
**42**	68.62	0.010962	PCa
